# Clinical and Histopathological Correlates of Endometrial Proliferative Lesions in Perimenopausal Women: A Retrospective Study with Internal Validation of a Risk Model

**DOI:** 10.3390/clinpract15100177

**Published:** 2025-09-26

**Authors:** Anca Daniela Brăila, Viorica Tudor, Cristian-Viorel Poalelungi, Constantin Marian Damian, Claudia Florina Bogdan-Andreescu, Alexandru Burcea, Andreea-Mariana Bănățeanu, Emin Cadar, Cristina-Crenguţa Albu

**Affiliations:** 1 Department of Obstetrics and Gynecology, University of Medicine and Pharmacy of Craiova, 200349 Craiova, Romania; anca.braila@umfcv.ro; 2 Integrated Outpatient Department of Obstetrics and Gynecology, “Prof. Dr. Agrippa Ionescu” Clinical Emergency Hospital, 011356 Bucharest, Romania; tudorviorica_med@yahoo.com; 3 Department of Obstetrics and Gynecology, “Carol Davila” University of Medicine and Pharmacy, 020021 Bucharest, Romania; 4 Department of Speciality Disciplines, “Titu Maiorescu” University, 031593 Bucharest, Romania; claudia.andreescu@prof.utm.ro (C.F.B.-A.); alexandru.burcea@prof.utm.ro (A.B.); andreea.banateanu@prof.utm.ro (A.-M.B.); 5 Faculty of Pharmacy, “Ovidius” University, 900470 Constanța, Romania; emin.cadar@365.univ-ovidius.ro; 6 Department of Genetics, “Carol Davila” University of Medicine and Pharmacy, 020021 Bucharest, Romania; cristina.albu@umfcd.ro

**Keywords:** endometrial hyperplasia, atypical hyperplasia, endometrial intraepithelial neoplasia, endometrial adenocarcinoma, perimenopause, biopsy curettage

## Abstract

**Background:** Endometrial proliferative lesions are common in the menopausal transition and carry a measurable risk of carcinoma. Early risk stratification may guide evaluation and follow-up. **Methods:** We performed a single-center retrospective study of 315 women aged 45–55 years (May 2021–May 2024) at a private clinic in Bucharest. Lesions were classified per WHO 2014 as hyperplasia without atypia, atypical hyperplasia/endometrial intraepithelial neoplasia (AH/EIN), or adenocarcinoma; “advanced pathology” was defined as AH/EIN or adenocarcinoma. Clinical comorbidities and transvaginal ultrasound endometrial thickness were recorded. Associations were tested with χ^2^; odds were estimated with multivariable logistic regression (adjusted ORs), with a modified Poisson sensitivity analysis for adjusted relative risk. Thickness differences were compared by one-way ANOVA, and severity correlations by Spearman’s ρ. Internal validation used 1000-bootstrap resampling. **Results:** Hyperplasia without atypia comprised 74.6% of cases, AH/EIN 20.0%, and adenocarcinoma 5.4% (advanced pathology 25.4%). Diabetes was independently associated with advanced pathology (aOR 2.75; 95% CI 1.14–6.61; *p* = 0.0237), while a history of non-atypical hyperplasia was inversely associated (aOR 0.31; 95% CI 0.13–0.72; *p* = 0.0068). Obesity showed a borderline association (aOR 1.79; 95% CI 0.98–3.26; *p* = 0.058), and long-term oral contraceptive use also approached significance (aOR 0.42; 95% CI 0.18–1.00; *p* = 0.051). Endometrial thickness increased stepwise with histopathological severity (ANOVA *p* < 0.0001; η^2^ = 0.44) and correlated with ordered severity (ρ = 0.634). The multivariable model showed moderate discrimination (AUC 0.68; optimism-corrected 0.66) with acceptable calibration (slope 0.92; Hosmer–Lemeshow *p* = 0.052) and overall accuracy (Brier 0.18). **Conclusions:** In perimenopausal abnormal bleeding, metabolic comorbidities—especially diabetes—together with increased endometrial thickness identify women at higher risk of AH/EIN or carcinoma. Histopathology remains the diagnostic reference. The model can aid clinical prioritization but requires external validation and should not be used as the sole basis for decisions.

## 1. Introduction

The endometrium is a hormonally regulated tissue, serving as a peripheral target for sex steroid hormones—primarily estrogens and progesterone—which govern its cyclical, biphasic transformation throughout the menstrual cycle. During the proliferative phase, estrogen stimulates uniform growth of the endometrial epithelium and stroma, increasing its thickness from approximately 1 mm in the early postmenstrual stage to 5–10 mm by the end of the secretory phase, which is coordinated by both estrogen and progesterone [1]. Throughout this process, the gland-to-stroma ratio remains balanced, and the tissue overall architecture is harmonious both macroscopically and microscopically, reflecting a physiologically functional endometrial environment [1].

Etymologically, the term *hyperplasia* derives from the Greek *hyper* (“more”) and *plassein* (“to form”) and refers to the abnormal proliferation of a single cell line, originating from a common cellular precursor [2,3,4]. Structurally, the endometrium comprises two main cell lines—epithelial and stromal—organized in two distinct architectural components: the glands and the cytogenic stroma [5,6]. Excessive estrogen stimulation, especially when unopposed by progesterone, disrupts this balance and results in histological patterns characteristic of endometrial hyperplasia (EH).

The 1994 World Health Organization (WHO) classification system divided EH into four categories based on architectural complexity and the presence of cytological atypia: simple or complex, each with or without atypia [7,8]. In 2014, the WHO introduced a simple and clinically relevant two-tier system: endometrial hyperplasia without atypia (a benign lesion with low malignant potential) and endometrial hyperplasia with atypia, also termed endometrial intraepithelial neoplasia (EIN), which represents a premalignant stage [6].

Endometrial carcinoma (EC), particularly the endometrioid subtype, is the most common gynecological malignancy in developed countries. Over 90% of cases occur in 50 plus women, with a mean age at diagnosis of approximately 63 years. Although rare before age 40, early-onset EC poses challenges for fertility preservation [9,10]. In the United States, endometrial cancer ranks first among gynecological malignancies, with an incidence of 0.3%, increasing to 0.5% when adjusted for hysterectomy rates [11,12]. In Romania, it ranks fourth in terms of gynecologic cancer incidence (7.8%) and accounts for approximately 5.9% of related mortality [13].

Endometrial cancer (EC) is histologically and molecularly classified into two distinct subtypes. Type I EC is estrogen-dependent, generally well-differentiated, and frequently appears from atypical endometrial hyperplasia. It is commonly associated with specific genetic alterations, including mutations in PTEN, PIK3CA, KRAS, and CTNNB1, as well as MLH1 promoter hypermethylation [14,15,16]. Conversely, type II EC tumors are estrogen-independent, poorly differentiated, and often display serous or clear cell histology, with frequent TP53 mutations [17].

The perimenopause is characterized by fluctuating estrogen levels and an early, constant decline in progesterone production due to anovulatory cycles. This hormonal imbalance creates a state of relative estrogen dominance, which can favor endometrial proliferation and result in irregular or heavy menstrual bleeding. Hyperestrogenism during this period may be absolute, due to factors such as exogenous hormone use or estrogen-secreting tumors, or relative, stemming from corpus luteum insufficiency and the anovulatory cycles that are typical of perimenopause [18].

Because early endometrial hyperplasia (EH) often develops without symptoms, it can go unnoticed until it progresses. Since it has the potential to turn malignant, identifying it early—through biopsy and histopathological analysis—remains the most reliable method of diagnosis [18].

This study aims to analyze the clinical and histopathological features of endometrial proliferative lesions in perimenopausal women and to develop a preliminary risk stratification model for endometrial adenocarcinoma. This model is designed to enable early detection of high-risk patients and to assist clinicians in providing individualized monitoring and treatment strategies.

## 2. Materials and Methods

### 2.1. Study Design and Setting

This retrospective, observational study was conducted over three years period (May 2021–May 2024) at a private Obstetrics and Gynecology clinic in Bucharest, Romania. The study was conducted in accordance with the Declaration of Helsinki and was approved by the Ethics Committee of Alco San Medical Center, Bucharest, Romania (protocol code 49; approval date 19 April 2021).

### 2.2. Study Population

The study included 315 female patients, aged between 45 and 55 years, corresponding to the menopausal transition period. The annual distribution of cases was as follows: 107 in 2021, 103 in 2022, and 105 in 2023. For analytical purposes, patients were divided into two age groups: 45–50 years and 51–55 years.

### 2.3. Data Sources and Variables

Clinical data were obtained from the clinic’s medical records, including age, personal medical history, and associated gynecological and breast pathology. Particular attention was given to comorbid conditions commonly related to endometrial pathology, such as obesity, diabetes mellitus, and cardiovascular diseases (primarily arterial hypertension). Gynecological comorbidities were also documented, including uterine fibroids, estrogen-secreting ovarian tumors, polycystic ovary syndrome (PCOS), and infertility, along with the presence of fibrocystic breast disease. Medication exposures recorded a priori included long-term oral contraceptive use and estrogen replacement therapy.

### 2.4. Clinical Presentation and Ultrasound Assessment

The main clinical indication for evaluation was abnormal uterine bleeding, which was the main symptom reported. However, a significant number of patients were asymptomatic, and lesions were incidentally detected during routine gynecological evaluations. In symptomatic cases, the bleeding patterns such as menorrhagia and menometrorrhagia were frequently observed, especially in early perimenopause, and were presumed to be related to luteal phase insufficiency and progesterone deficit. All patients underwent a standardized diagnostic protocol. Hematological investigations and urinalysis were performed as part of the initial evaluation. Imaging included both transabdominal and transvaginal ultrasonography to assess endometrial thickness and to identify associated pelvic pathology.

### 2.5. Endometrial Sampling and Histopathology

Specific diagnostic measures for endometrial proliferative lesions included fractional endometrial biopsy curettage (with separate endocervical and endometrial sampling where indicated), followed by histopathological examination of hematoxylin-and-eosin (H&E)–stained sections. Hysteroscopy was used selectively to evaluate focal intrauterine abnormalities and to facilitate targeted biopsy of suspected lesions, such as endometrial polyps, hyperplastic areas, or suspected neoplastic growths.

Histopathological classification. For primary reporting and analysis, all lesions were classified according to the WHO 2014 two-tier system—hyperplasia without atypia and atypical hyperplasia/endometrial intraepithelial neoplasia (AH/EIN); endometrial adenocarcinoma was reported separately. Because some original pathology reports used legacy 1994 terminology, we reclassified those entries as follows: simple hyperplasia and complex hyperplasia without atypia were reclassified as hyperplasia without atypia; complex atypical hyperplasia and EIN were reclassified as AH/EIN. The detailed mapping is provided in Supplementary Table S1, and extended prevalence tables appear in Supplementary Table S2.

### 2.6. Microphotography and Image Processing

Hematoxylin–eosin (H&E) microphotographs were acquired using a bright-field light microscope equipped with a digital camera at magnifications of ×4, ×10, and ×20. Scale bars (500 µm for ×4, 200 µm for ×10, 100 µm for ×20) were calibrated with a stage micrometer and overlaid in the exported images. Images were saved as TIFF (600 dpi). Only global, linear adjustments of white balance and brightness/contrast were applied uniformly across the whole image; no local, selective edits, sharpening, or content-altering manipulations were performed. Panel labels (a–d) were added for orientation to highlight the relevant morphological features.

These micrographs are provided for illustrative purposes only to demonstrate representative morphology for each diagnostic category; no quantitative measurements or outcomes were derived from these images.

### 2.7. Advanced Imaging

In selected cases where clinical or histological findings raised suspicion of malignancy or necessitated surgical planning, additional imaging was performed using computed tomography (CT) and magnetic resonance imaging (MRI). Although these modalities provide valuable diagnostic information, their limited accessibility and high cost make them unsuitable for population-wide screening.

### 2.8. Statistical Analysis

Descriptive statistics were computed for demographic and clinical variables. Categorical variables are reported as counts and percentages; continuous variables as mean ± standard deviation or median (interquartile range), according to distribution (assessed with the Shapiro–Wilk and Levene tests).

Primary inferential analyses (WHO 2014 framework). Associations between categorical predictors (obesity, hypertension, diabetes, PCOS, infertility, uterine fibroids/polyps, fibrocystic breast disease, estrogen-producing ovarian tumors, long-term oral contraceptive use, estrogen replacement therapy, and history of non-atypical hyperplasia) and diagnostic grouping were evaluated using Pearson’s χ^2^ test (or Fisher’s exact test where appropriate), with two-sided *p* values and Cramér’s *V* as effect size. For clinical interpretability, a binary outcome was defined: “advanced pathology” (AH/EIN or adenocarcinoma) vs. “non-advanced” (hyperplasia without atypia). Logistic regression was used to estimate odds ratios (ORs) with 95% confidence intervals (CIs). A multivariable model also included age group and all comorbidities to obtain adjusted ORs (aORs). For logistic and modified Poisson regressions, we present effect estimates with 95% confidence intervals and two-sided Wald *p*-values for all predictors; statistical significance was set at *α* = 0.05 after FDR control.

Sensitivity analysis (relative risk). Because the outcome prevalence was ~25%, we estimated relative risks (RR) for advanced pathology using modified Poisson regression with robust (sandwich) variances, adjusted for the same covariates as the multivariable logistic model. As a sensitivity analysis, we also fit a modified Poisson regression with robust (sandwich) variances; conclusions were unchanged relative to the logistic model.

Continuous variables. Group differences in endometrial thickness were assessed by one-way ANOVA (or Kruskal–Wallis when assumptions were violated), with Tukey’s HSD post hoc tests.

Correlation analyses. Spearman’s rank correlation was used to assess the associations between an ordinal histopathological severity score (0–4: simple hyperplasia to carcinoma) and clinical variables, including endometrial thickness and a metabolic index (sum of obesity, hypertension, and diabetes).

Multiple testing and thresholds. Multiple testing was controlled using the Benjamini–Hochberg false discovery rate. All tests were two-sided with *α* = 0.05.

Internal validation. To quantify potential overfitting, we performed bootstrap internal validation with 1000 stratified resamples (sampling with replacement from the original cohort, preserving the proportion of advanced vs. non-advanced outcomes). At each iteration, the full multivariable model was refitted in the bootstrap sample and evaluated both in the bootstrap sample (apparent performance) and in the original sample. Optimism was estimated as the average difference and subtracted from the apparent metrics to obtain optimism-corrected performance. We report the optimism-corrected AUC (ROC), Brier score, and calibration intercept/slope (the latter from regressing observed outcomes on predicted log-odds).

Analyses were performed in IBM SPSS Statistics v28 (IBM Corp., Armonk, NY, USA) and independently replicated in Python (statsmodels v0.13, SciPy v1.11) to ensure reproducibility.

## 3. Results

### 3.1. Patient Characteristics

**Figure 1:**
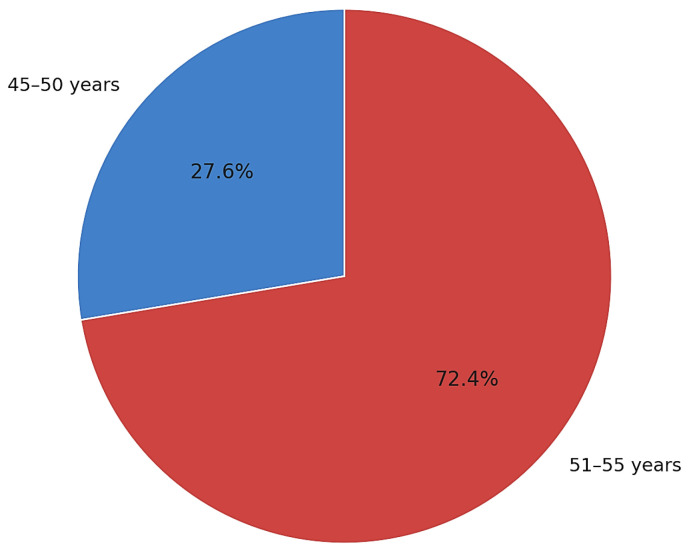
Distribution of patients by age group (*n* = 315): 45−50 years, 27.6%; 51−55 years, 72.4%.

**Figure 2:**
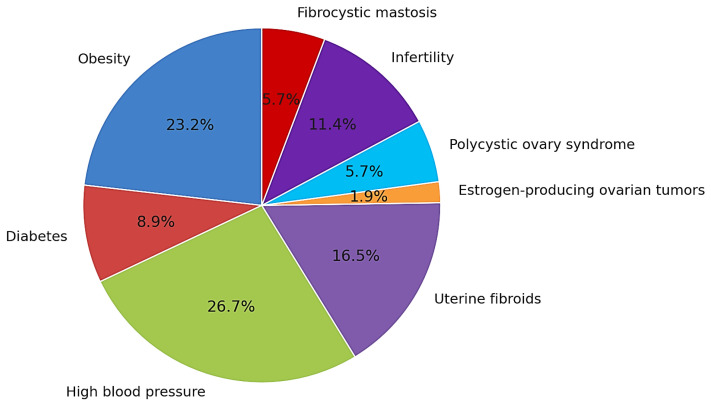
Distribution of associated comorbidities in patients diagnosed with endometrial proliferative lesions; most prevalent: cardiovascular disease (26.7%), obesity (23.2%), uterine fibroids (16.5%), and infertility (11.4%).

Baseline characteristics are summarized in Table 1. Of the 315 patients aged 45–55 years, 87 (27.6%) were 45–50 years old and 228 (72.4%) were 51–55 years old (Figure 1). Comorbidities and ultrasound findings are detailed in Table 1; the distribution of comorbidities is visualized in Figure 2.

### 3.2. WHO 2014 Classification of Endometrial Lesions

Using the WHO 2014 scheme, the distribution of lesions was as follows: hyperplasia without atypia—235/315 (74.6%; 95% CI, 69.5–79.1); atypical hyperplasia/EIN (AH/EIN)—63/315 (20.0%; 95% CI, 16.0–24.8); and endometrial adenocarcinoma—17/315 (5.4%; 95% CI, 3.4–8.5). For clinical context, advanced lesions (AH/EIN/adenocarcinoma) comprised 80/315 (25.4%; 95% CI, 20.9–30.5), (Table 2).

**Figure 3:**
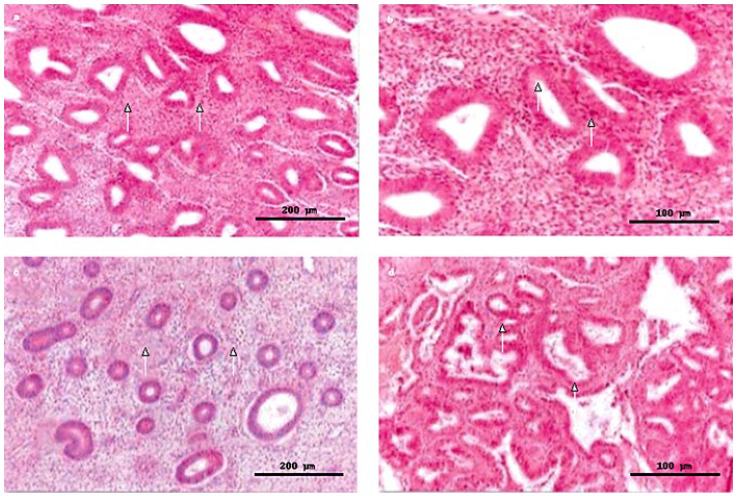
Endometrial hyperplasia without atypia (WHO 2014; Hematoxylin & Eosin stain). Images were acquired using bright-field microscopy at 10× or 20× magnification; scale bars were calibrated with a stage micrometer (200 µm for 10×; 100 µm for 20×). (**a**,**c**) Representative fields showing hyperplastic endometrial glands with mild architectural irregularities, lined by a single layer of benign epithelial cells, and maintaining an approximately equal gland-to-stroma ratio (H&E, 10×; scale bar = 200 µm; white arrows indicate interglandular stroma). (**b**) Higher magnification view illustrating glands lined by a single epithelial layer with monomorphic nuclei and mild anisokaryosis, without cytological atypia (H&E, 20×; scale bar = 100 µm; white arrows indicate a representative gland and adjacent interglandular stroma). (**d**) High-power field of endometrial hyperplasia without atypia, showing increased glandular proliferation with a gland-to-stroma ratio shifted in favor of glands, yet still lined by a single layer of epithelial cells with monomorphic nuclei (H&E, 20×; scale bar = 100 µm; white arrows highlight proliferating glands).

**Figure 4:**
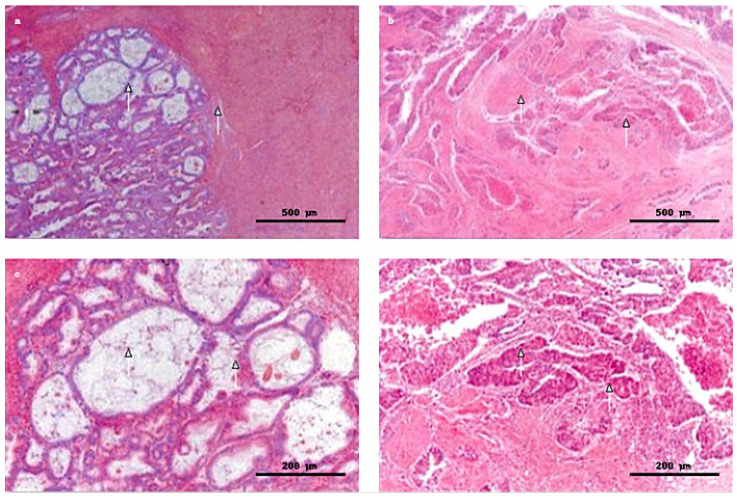
Representative morphologies of endometrial adenocarcinoma (Hematoxylin & Eosin stain). Images were acquired using bright-field microscopy at 4× or 10× magnification; scale bars were calibrated with a stage micrometer (500 µm for 4×; 200 µm for 10×). (**a**,**b**) Low-power views of endometrial intraepithelial neoplasia (EIN)/atypical hyperplasia, showing crowded and confluent hyperplastic glands separated by minimal stroma, with back-to-back glandular architecture. Glands are lined by atypical epithelium, composed of multilayered epithelial cells with vesicular nuclei, anisokaryosis, and anisokaryochromia (H&E, 4×; scale bar = 500 µm; white arrows highlight confluent glandular structures and reduced stromal component). (**c**) Intermediate-power view demonstrating progression toward invasive adenocarcinoma, with a cribriform pattern, variable gland size and shape, and more pronounced cytological atypia (H&E, 10×; scale bar = 200 µm; white arrows indicate enlarged atypical glands). (**d**) High-power field of invasive endometrial adenocarcinoma, showing loss of glandular polarity, stromal invasion, epithelial malignant buds infiltrating the stroma, and areas of tumor necrosis (H&E, 10×; scale bar = 200 µm; white arrows mark malignant epithelial buds invading the stroma).

Representative histopathology is shown in Figure 3 and Figure 4: hyperplasia without atypia (Figure 3) and endometrial adenocarcinoma (Figure 4).

For surgical planning in selected cases (AH/EIN or confirmed malignancy), advanced CT/MRI was used as clinically indicated.

### 3.3. Distribution of Clinical Risk Factors Across Histopathological Diagnoses

The crude distribution of risk factors by histopathological category is shown in Table 3. Obesity, hypertension, and diabetes were numerically more frequent in advanced lesions (AH/EIN/adenocarcinoma). In contrast, fibroids/polyps, fibrocystic breast disease, PCOS, and estrogen-dependent tumors showed no progressive gradient across severity.

### 3.4. Clinicopathological Associations Across Histopathological Categories (χ^2^)

Significant associations with diagnostic category were observed for obesity (χ^2^ = 16.67, df = 4, *p =* 0.0022, Cramér’s *V* = 0.23), diabetes (χ^2^ = 11.30, df = 4, *p =* 0.0234, *V* = 0.19), hypertension (χ^2^ = 10.83, df = 4, *p =* 0.0285, *V* = 0.19), and history of non-atypical hyperplasia (χ^2^ = 11.26, df = 4, *p =* 0.0238, *V* = 0.19). Infertility, fibroids/polyps, fibrocystic breast disease, PCOS, estrogen-producing tumors, estrogen replacement therapy, and long-term oral contraceptives were not significant (Table 4).

### 3.5. Predictors of Advanced Endometrial Pathology: Univariable Logistic Regression

(“Advanced” was defined as AH/EIN/adenocarcinoma; reference group = hyperplasia without atypia.)

In univariable models contrasting advanced vs. non-advanced lesions, diabetes (OR = 2.85, 95% CI 1.29–6.28, *p =* 0.0096) and obesity (OR = 1.93, 95% CI 1.09–3.39, *p =* 0.023) were associated with higher odds, whereas history of non-atypical hyperplasia (OR = 0.29, 95% CI 0.13–0.67, *p =* 0.0037) and long-term oral contraceptives (OR = 0.38, 95% CI 0.17–0.89, *p =* 0.0251) were protective (Table 5).

### 3.6. Predictors of Advanced Endometrial Pathology: Multivariable Logistic Regression

After adjustment for age group and comorbidities, diabetes was independently associated with advanced pathology (aOR = 2.75, 95% CI 1.14–6.61, *p =* 0.0237), whereas history of non-atypical hyperplasia was protective (aOR = 0.31, 95% CI 0.13–0.72, *p =* 0.0068). Obesity (aOR = 1.79, 95% CI 0.98–3.26, *p =* 0.058) and long-term oral contraceptives (aOR = 0.42, 95% CI 0.18–1.00, *p =* 0.051) were borderline (Table 6).

Model diagnostics. No multicollinearity was detected (all variance inflation factors < 2), and no unduly influential observations were identified (maximum Cook’s distance < 1; |standardized residuals| ≤ 3).

Sensitivity analysis (relative risk). Using modified Poisson regression with robust variances, effect estimates were similar, and statistical significance was unchanged.

### 3.7. Endometrial Thickness Across Histopathological Diagnoses

Endometrial thickness differed significantly across diagnostic categories (F(4, 310) = 61.33, *p* < 0.0001), with a large effect size (*η^2^* = 0.44). Tukey’s HSD confirmed a graded increase from simple hyperplasia to carcinoma, with significant pairwise contrasts for most adjacent categories (carcinoma vs. complex atypical: mean difference 2.63 mm, *p =* 0.0014; complex atypical vs. complex without atypia: 2.02 mm, *p =* 0.0002; complex without atypia vs. simple: 1.98 mm, *p* < 0.0001). EIN was thicker than complex without atypia and simple (both *p* < 0.0001), but not significantly different from carcinoma or complex atypical. The ANOVA summary is shown in Table 7. Detailed pairwise post hoc comparisons (Tukey’s HSD) are presented in Supplementary Table S3.

### 3.8. Apparent Performance of the Multivariable Model

In the derivation (apparent) analysis, the multivariable logistic regression model showed moderate discrimination (AUC = 0.68). Calibration by the Hosmer–Lemeshow goodness-of-fit test was acceptable (χ^2^ = 15.4, df = 8, *p* = 0.052), indicating no significant deviation between predicted and observed risks across deciles. The Nagelkerke R^2^ was 0.14, suggesting that ≈approximately 14% of the variance in advanced pathology is explained by the included predictors. Summary metrics are reported in Table 8. and statistical significance was unchanged. For transparency, we report two-sided *p*-values alongside 95% CIs for all regression coefficients; the Hosmer–Lemeshow goodness-of-fit test yielded χ^2^ = 15.4 (df = 8), *p* = 0.052.

### 3.9. Clinicopathological Correlations with Histopathological Severity

Spearman rank correlations with ordered histopathological severity (0–4) showed a strong positive association with endometrial thickness (ρ = 0.634, *p* ≈ 7.6 × 10^−37^) and a modest positive association with a metabolic index (obesity + hypertension + diabetes; ρ = 0.253, *p =* 5.6 × 10^−6^). The overall risk count (sum of all factors) was not significant (ρ = 0.064, *p =* 0.254). At the individual level, obesity (ρ = 0.184, *p =* 0.0010), hypertension (ρ = 0.154, *p =* 0.0061), and diabetes (ρ = 0.125, *p =* 0.0268) correlated positively with severity; history of non-atypical hyperplasia (ρ = −0.139, *p =* 0.0137) and long-term oral contraceptives (ρ = −0.126, *p =* 0.0255) correlated negatively. Infertility, fibroids/polyps, fibrocystic breast disease, PCOS, estrogen-producing tumors, and estrogen replacement therapy showed no significant correlations. Full correlation matrices and variable-level coefficients are available in Supplementary Tables S4 and S5.

### 3.10. Internal Validation of the Multivariable Model

We performed bootstrap internal validation (Harrell’s approach) with 1000 stratified resamples. In each resample the full model was refit; optimism was estimated as the mean difference between the bootstrap apparent and test performance and subtracted from the model’s apparent performance. The optimism-corrected discrimination was AUC = 0.66 (apparent AUC = 0.68). Calibration remained acceptable with a calibration slope = 0.92 (heuristic shrinkage) and calibration intercept ≈ 0.01, and overall accuracy was Brier score = 0.18. These findings indicate moderate discrimination with acceptable calibration after correction for optimism (Table 9).

## 4. Discussion

The menopausal transition is a progressive physiological process that marks the shift from the reproductive phase, defined by regular menstrual cycles and ovulation, to ovarian senescence and the permanent cessation of menstruation. This transitional period usually extends 4 to 7 years, beginning with menstrual irregularity and ending one year after the last menstrual period [19].

In early menopausal transition, follicle-stimulating hormone (FSH) levels rise modestly due to a decline in ovarian inhibin secretion, which exerts typically negative feedback on the hypothalamic-pituitary axis [20,21]. During the late transition phase, folliculogenesis becomes increasingly erratic, leading to anovulation. As ovarian follicular reserves become depleted, inhibin levels diminish, and FSH secretion increases further [22,23].

The anti-Müllerian hormone (AMH), secreted by granulosa cells of secondary and preantral follicles, serves as a marker of ovarian reserve and decreases progressively during the perimenopausal period [24,25]. With the cessation of ovarian steroidogenesis, circulating levels of FSH and luteinizing hormone (LH) increase, indicating ovarian failure. The primary mechanism leading the transition to menopause is follicular atresia.

Microscopic endometrial alterations reflect accurately the hormonal fluctuations characteristic of the menopausal transition. In early perimenopause, endometrial morphology often continues to exhibit features of ovulatory cycles. As perimenopause progresses, however, anovulation becomes more frequent, leading to unopposed estrogen stimulation and resulting in proliferative or even dysplastic endometrial changes. Following menopause, hypoestrogenism predominates, and the endometrium becomes atrophic. Endometrial proliferative lesions are more prevalent during the menopausal transition and are influenced by both endocrine and genetic factors [26]. In the literature, reported incidence ranges from 48.5% to 83.8% [27,28,29,30]. In our cohort, hyperplasia without atypia accounted for 74.6% of cases, atypical hyperplasia/EIN (AH/EIN) for 20.0%, and endometrial adenocarcinoma for 5.4%; accordingly, “advanced” pathology (AH/EIN or carcinoma) comprised 25.4%, in line with prior studies [31]. While some lesions are initially functional, reflecting the hormonal changes characteristic of perimenopause, other lesions, like AH/EIN and carcinoma, represent severe pathological developments that require immediate intervention.

### 4.1. Principal Findings

Across the WHO-2014 histopathological categories, the prevalence of obesity, diabetes, and hypertension increased with diagnostic severity (χ^2^ significant; Cramér’s V ≈ 0.19–0.23). In the multivariable analysis, diabetes remained an independent predictor of advanced pathology (aOR 2.75; 95% CI 1.14–6.61; *p* = 0.0237), while a history of non-atypical hyperplasia was protective (aOR 0.31; 95% CI 0.13–0.72; *p* = 0.0068). Obesity (aOR 1.79; 95% CI 0.98–3.26; *p* = 0.058) and long-term oral contraceptive use (aOR 0.42; 95% CI 0.18–1.00; *p* = 0.051) showed borderline associations. Results were similar when expressed as adjusted relative risks using modified Poisson regression with a robust variance estimator.

### 4.2. Ultrasound–Pathology Concordance

Endometrial thickness increased monotonically with histopathological severity (ANOVA *p* < 0.0001; large effect size, η^2^ = 0.44), and post hoc contrasts confirmed step-ups across adjacent categories. Spearman correlations supported a strong association between endometrial thickness and ordered severity (ρ = 0.634) and a modest positive association for a metabolic index (obesity + hypertension + diabetes; ρ = 0.253), consistent with a metabolic–estrogenic drive in endometrial carcinogenesis.

### 4.3. Predictive Performance and Internal Validation

The multivariable model showed moderate apparent discrimination (AUC 0.68) and acceptable calibration (Hosmer–Lemeshow *p* = 0.052; Nagelkerke R^2^ = 0.14). Bootstrap internal validation (1000 resamples) indicated modest optimism (optimism-corrected AUC 0.66), with a calibration slope of 0.92 (heuristic shrinkage) and Brier score 0.18, suggesting the model may serve as a supportive risk indicator for clinical prioritization rather than a standalone triage tool.

### 4.4. Biological Plausibility and Molecular Context

EC is genetically heterogeneous and classified into two major types based on histologic, clinical, and molecular features: Type I (endometrioid) and Type II (non-endometrioid) carcinomas [32,33,34]. Type I EC is frequently associated with mutations in PTEN, KRAS, CTNNB1, PIK3CA, and MLH1 promoter hypermethylation [33]. Among these, PTEN, located on chromosome 10q23, is the most commonly mutated tumor suppressor gene in endometrioid EC, present in 57–83% of cases and even in 10% of Type II ECs [35].

PTEN regulates cell proliferation by arresting the cell cycle at the G1/S checkpoint, inhibiting cell migration, adhesion, and downstream mitogenic pathways such as MAPK signaling [36]. It also modulates both pro-apoptotic and anti-apoptotic signals [37]. PTEN inactivation results in uncontrolled cellular proliferation and survival and may represent an early event in endometrial carcinogenesis, often in the context of prolonged unopposed estrogen exposure [38]. Loss of PTEN expression has been reported in 55% of atypical hyperplasia cases and 83% of endometrial carcinomas [39].

Microsatellite instability (MSI), another genetic hallmark, is observed in early carcinogenic stages in a subset of ECs [40]. Type II ECs, particularly serous carcinomas, exhibit frequent TP53 mutations, present in over 90% of these tumors and approximately 17% of endometrioid variants [41,42].

### 4.5. Clinical and Diagnostic Implications

From a clinical perspective, careful administration of estrogen in hormone replacement therapy or oral contraceptives is necessary. However, estrogen supplementation without adequate progesterone to balance endometrial changes increases the risk of endometrial proliferation and neoplasia.

Paraclinical evaluation via hysteroscopy, transvaginal ultrasonography, and imaging (CT, MRI) remains routine for diagnosis and monitoring of endometrial lesions [43,44,45,46]. But, the gold standard for early detection of endometrial dysplasia remains biopsy curettage followed by histopathological assessment [47].

### 4.6. Exploratory Risk Index

Any equal-weight composite derived from these predictors should be considered exploratory. External validation is required before clinical use; until then, such indices should not be used as the sole basis for clinical decision-making.

## 5. Limitations

The single-center, retrospective nature of this study may limit generalizability and is susceptible to selection and information biases. Although we applied WHO 2014 classifications uniformly, with transparent mapping from legacy terminology, some misclassification in historical reports is possible. The model explains a modest proportion of variance (Nagelkerke R^2^ = 0.14) and, despite bootstrap validation, requires external validation. We did not include centralized pathology review, granular metabolic profiling, or core-lab imaging standardization.

## 6. Suggestions for Future Research

Future research should focus on

- Validating the model externally across centers and care settings;
- Assessing calibration and progression endpoints longitudinally;
- Testing augmentation with quantitative ultrasound parameters, refined metabolic markers, and molecular features;
- Performing decision-curve analysis to evaluate clinical utility.

## 7. Conclusions

In this cohort of 315 women aged 45–55 years, hyperplasia without atypia accounted for 74.6% of lesions, atypical hyperplasia/EIN for 20.0%, and endometrial adenocarcinoma for 5.4%; advanced pathology (AH/EIN or carcinoma) comprised 25.4%. Histopathology remains the reference standard for diagnosis and risk stratification.

Diabetes was independently associated with advanced pathology (aOR 2.75; 95% CI 1.14–6.61), whereas a history of non-atypical hyperplasia was inversely associated (aOR 0.31; 95% CI 0.13–0.72). Obesity showed a borderline association. Endometrial thickness increased stepwise with histopathological severity and correlated strongly with ordered severity.

The multivariable model showed moderate discrimination and acceptable calibration (AUC 0.68; optimism-corrected AUC 0.66). External validation is required before clinical use; the model should not be used as the sole basis for decisions. Clinically, perimenopausal patients with abnormal bleeding, metabolic comorbidities like diabetes and/or obesity, and increased endometrial thickness should be prioritized for endometrial sampling and close follow-up.

## Figures and Tables

**Table 1:** Baseline characteristics of the cohort (*n* = 315).

Characteristic	*n* (%) or Summary
**Age group, years**	
45–50	87 (27.6)
51–55	228 (72.4)
**Systemic comorbidities**	
Cardiovascular disease (primarily hypertension)	84 (26.7)
Obesity	73 (23.2)
Diabetes mellitus	28 (8.9)
**Gynecologic/breast comorbidities**	
Uterine fibroids/polyfibromatosis	52 (16.5)
Polycystic ovary syndrome (PCOS)	18 (5.7)
Infertility	36 (11.4)
Fibrocystic breast disease	18 (5.7)
Estrogen-secreting ovarian tumors	6 (1.9)
**Ultrasound findings**	
Endometrial thickness, mm	range 5–17
Cystic/polypoid structures (suggestive of polyps)	32 (10.2)
Heterogeneous endometrial architecture (suspicious for carcinoma)	12 (3.8)

Note. Percentages are column percentages based on *n* = 315. Cardiovascular disease refers predominantly to high blood pressure, as recorded in the charts.

**Table 2:** WHO 2014 classification of endometrial lesions (*n* = 315).

Category	*n*/N	% (95% CI)
Hyperplasia without atypia	235/315	74.6 (69.5–79.1)
Atypical hyperplasia/EIN (AH/EIN)	63/315	20.0 (16.0–24.8)
Endometrial adenocarcinoma	17/315	5.4 (3.4–8.5)

Note. Values are *n*/N and % (95% CI); confidence intervals use the exact (Wilson) method. Advanced lesions for downstream analyses are defined as AH/EIN or adenocarcinoma (combined prevalence 25.4%; 95% CI, 20.9–30.5). Extended prevalence tables are provided in Supplementary Table S2.

**Table 3:** Distribution of risk factors across histopathological diagnoses (values are *n*, % within diagnostic group).

Risk Factor	Simple Hyperplasia	Complex Hyperplasia (No Atypia)	Complex Atypical Hyperplasia	EIN	Adenocarcinoma	Total (%)
Obesity	24 (14.7%)	23 (31.9%)	16 (41.0%)	6 (25.0%)	4 (23.5%)	73 (23.2%)
Hypertension	34 (20.9%)	22 (30.6%)	10 (25.6%)	9 (37.5%)	9 (52.9%)	84 (26.7%)
Diabetes	11 (6.7%)	4 (5.6%)	4 (10.3%)	6 (25.0%)	3 (17.6%)	28 (8.9%)
Infertility	18 (11.0%)	8 (11.1%)	6 (15.4%)	2 (8.3%)	2 (11.8%)	36 (11.4%)
Fibroids/Polyps	29 (17.8%)	13 (18.1%)	6 (15.4%)	3 (12.5%)	1 (5.9%)	52 (16.5%)
Fibrocystic breast disease	7 (4.3%)	7 (9.7%)	2 (5.1%)	1 (4.2%)	1 (5.9%)	18 (5.7%)
PCOS	9 (5.5%)	5 (6.9%)	2 (5.1%)	1 (4.2%)	1 (5.9%)	18 (5.7%)
Estrogen-producing tumors	3 (1.8%)	2 (2.8%)	1 (2.6%)	0 (0.0%)	0 (0.0%)	6 (1.9%)

Note. Legacy (1994) subtypes are shown for descriptive continuity only; all inferential analyses use the WHO 2014 framework.

**Table 4:** Chi-square tests: association of categorical clinical factors with histopathological diagnosis categories.

Factor	Chi-Square	df	*p*-Value	Cramér’s *V*
Obesity	16.67	4	0.0022	0.23
Hypertension	10.83	4	0.0285	0.19
Diabetes	11.30	4	0.0234	0.19
History of non-atypical hyperplasia	11.26	4	0.0238	0.19
Oral contraceptives (long-term)	5.76	4	0.2180	0.14
Fibrocystic breast disease	2.89	4	0.5766	0.10
Fibroids/Polyps	2.03	4	0.7306	0.08
Estrogen-producing tumors	1.18	4	0.8807	0.06
Estrogen replacement therapy	1.23	4	0.8731	0.06
Infertility	0.86	4	0.9298	0.05
PCOS	0.35	4	0.9867	0.03

Note. Pearson’s χ^2^ (Fisher’s exact where appropriate); two-sided *p*; Cramér’s *V* as effect size.

**Table 5:** Predictors of advanced endometrial pathology (univariable logistic regression).

Factor	OR	95% CI (Low–High)	*p*-Value
Diabetes	2.85	1.29–6.28	0.0096
Obesity	1.93	1.09–3.39	0.0230
History of non-atypical hyperplasia	0.29	0.13–0.67	0.0037
Oral contraceptives (long-term)	0.38	0.17–0.89	0.0251
Hypertension	1.72	0.99–2.98	0.0525
Fibroids/Polyps	0.66	0.31–1.38	0.2662
Estrogen replacement therapy	1.18	0.56–2.49	0.6671
PCOS	0.83	0.27–2.60	0.7503
Fibrocystic breast disease	0.83	0.27–2.60	0.7503
Estrogen-producing tumors	0.58	0.07–5.06	0.6240
Infertility	1.15	0.53–2.50	0.7275

Note. Univariable logistic regression (logit link) for advanced vs. non-advanced pathology. OR > 1 indicates higher odds of advanced pathology; OR < 1 indicates lower odds (protective). Two-sided Wald *p*. Abbreviations: PCOS, polycystic ovary syndrome.

**Table 6:** Multivariable logistic regression: adjusted odds ratios (aOR), 95% confidence intervals, and two-sided *p*-values for advanced vs. non-advanced endometrial pathology.

Factor	aOR	95% CI (Low–High)	*p*-Value
Diabetes	2.75	1.14–6.61	0.0237
History of non-atypical hyperplasia	0.31	0.13–0.72	0.0068
Obesity	1.79	0.98–3.26	0.0582
Oral contraceptives (long-term)	0.42	0.18–1.00	0.0512
Hypertension	1.68	0.93–3.03	0.0838
Fibroids/Polyps	0.72	0.33–1.58	0.4096
Estrogen-producing tumors	0.43	0.05–4.12	0.4673
Fibrocystic breast disease	0.63	0.18–2.21	0.4749
Estrogen replacement therapy	1.25	0.55–2.83	0.5987
PCOS	0.73	0.22–2.41	0.6072
Infertility	1.08	0.47–2.48	0.8488
Age 51–55 vs. 45–50	0.97	0.52–1.81	0.9204

Note. Model includes age group and all listed comorbidities. Values are adjusted odds ratios (aOR) with 95% CIs and two-sided Wald *p*-values. No multicollinearity was detected (all VIF < 2); no influential outliers (max Cook’s D < 1; |standardized residuals| ≤ 3).

**Table 7:** One-way ANOVA for endometrial thickness across histopathological diagnosis categories.

Source	Sum of Squares	df	F	*p*-Value
Diagnosis	1357.77	4	61.33	<0.0001
Residual	1715.64	310		

Note. One-way ANOVA with Tukey’s HSD post hoc (see Supplementary Table S3). Effect size for the overall model: *η^2^* = 0.44 (large).

**Table 8:** Apparent performance metrics for the multivariable model.

Metric	Value	Interpretation
AUC (ROC)	0.68	Moderate discrimination
Hosmer–Lemeshow χ^2^ (df = 8), *p*	15.4, *p =* 0.052	Acceptable calibration (no lack-of-fit)
Nagelkerke R^2^	0.14	Modest explained variance

Note. “Apparent” performance reflects in-sample estimates prior to internal validation.

**Table 9:** Model performance: apparent and optimism-corrected estimates (bootstrap, 1000 resamples).

Metric	Apparent	Optimism-corrected
AUC (ROC)	0.68	0.66
Calibration slope	—	0.92
Calibration intercept	—	≈0.01
Brier score	—	0.18

*Notes:* Lower Brier score indicates better overall accuracy (range 0–1).

## Data Availability

The data presented in this study are available on request from the corresponding author.

## Supplementary Materials

The following supporting information can be downloaded at https://www.mdpi.com/article/10.3390/clinpract15100177/s1, Table S1: Mapping of historical 1994 categories to WHO 2014 classification (with examples). Table S2: Extended prevalence tables (stratified summaries and confidence intervals). Table S3: Tukey’s HSD pairwise comparisons for endometrial thickness across diagnostic categories. Table S4: Spearman correlations between individual clinical factors and ordered histopathological severity. Table S5: Correlations for composite indices (e.g., metabolic index) and sensitivity analyses.

## Abbreviations

The following abbreviations are used in this manuscript:

EH	endometrial hyperplasia
WHO	World Health Organization
EIN	endometrial intraepithelial neoplasia
EC	endometrial carcinoma, endometrial cancer
PCOS	polycystic ovary syndrome
H&E	hematoxylin-and-eosin
AH/EIN	atypical hyperplasia/endometrial intraepithelial neoplasia
CT	computed tomography
MRI	magnetic resonance imaging
ORs	odds ratios
CIs	confidence intervals
aORs	adjusted confidence intervals
AMH	anti-Müllerian hormone
LH	luteinizing hormone
MSI	microsatellite instability
