# Supplementary material for: Clinical and Histopathological Correlates of Endometrial Proliferative Lesions in Perimenopausal Women: A Retrospective Study with Internal Validation of a Risk Model
Source: Clin Pract. 2025 Sep 26;15(10):177. doi: 10.3390/clinpract15100177 (PMC12563367; doi:10.3390/clinpract15100177)
Supplement: Supplementary file 1 [file clinpract-15-00177-s001.zip › clinpract-3830073-supplementary.pdf]

clinics and practice

MDPI

Supplementary Materials

Article

Clinical and Histopathological Correlates of Endometrial Proliferative Lesions in Perimenopausal Women: A Retrospective Study with Internal Validation of a Risk Model

Anca Daniela Brăila [1,†], Viorica Tudor [2,†], Cristian-Viorel Poalelungi [3,*], Constantin Marian Damian [1,*], Claudia Florina Bogdan-Andreescu [4], Alexandru Burcea [4], Andreea-Mariana Bănățeanu [4], Emin Cadar [5] and Cristina-Crenguţa Albu [6]

Table S1. Mapping of historical 1994 categories to WHO 2014 classification (with examples)

Historical (1994) category	WHO 2014 mapped category
Simple hyperplasia	Hyperplasia without atypia
Complex hyperplasia (without atypia)	Hyperplasia without atypia
Atypical hyperplasia (simple/complex)	Atypical hyperplasia / EIN (AH/EIN)
Endometrial intraepithelial neoplasia (EIN)*	Atypical hyperplasia / EIN (AH/EIN)
Endometrial adenocarcinoma	Endometrial adenocarcinoma (reported separately)

Notes. WHO 2014 collapses simple/complex without atypia into Hyperplasia without atypia, and atypical hyperplasia (simple/complex) together with EIN into AH/EIN; adenocarcinoma remains a separate category. *EIN did not belong to the WHO-1994 scheme but appeared in some pathology reports and is mapped to AH/EIN under WHO-2014.

Table S2. Extended prevalence tables (stratified summaries and confidence intervals)

(A) WHO 2014 framework

Category	n / N	% (95% CI)
Hyperplasia without atypia	235 / 315	74.6 (69.5–79.1)
Atypical hyperplasia / EIN (AH/EIN)	63 / 315	20.0 (16.0–24.8)
Endometrial adenocarcinoma	17 / 315	5.4 (3.4–8.5)
Advanced lesions (AH/EIN + adenocarcinoma)	80 / 315	25.4 (20.9–30.5)

(B) Historical 1994 subcategories (for transparency)

Category	n / N	% (95% CI)
Simple hyperplasia	163 / 315	51.7 (46.2–57.2)
Complex hyperplasia (without atypia)	72 / 315	22.9 (18.6–27.8)
Complex atypical hyperplasia	39 / 315	12.4 (9.2–16.5)
Endometrial intraepithelial neoplasia (EIN)	24 / 315	7.6 (5.2–11.1)

Endometrial adenocarcinoma	17 / 315	5.4 (3.4–8.5)

Notes. Values are n/N and % (95% CI, Wilson exact). All inferential analyses in the main text use the WHO-2014 framework.

Table S3. Tukey's HSD pairwise comparisons for endometrial thickness across diagnostic categories

Pairwise contrast (A – B)	Mean difference (mm)	95% CI (mm)	Adjusted p
Complex (no atypia) – Simple	1.98	1.34 to 2.62	<0.0001
Complex atypical – Complex (no atypia)	2.02	0.98 to 3.06	0.0002
Carcinoma – Complex atypical	2.63	1.06 to 4.20	0.0014
EIN – Complex (no atypia)	1.85	0.93 to 2.77	<0.0001
EIN – Simple	3.83	2.96 to 4.70	<0.0001
Complex atypical – Simple	4.00	3.05 to 4.95	<0.0001
Carcinoma – Complex (no atypia)	4.65	3.22 to 6.08	<0.0001
Carcinoma – Simple	6.63	5.29 to 7.97	<0.0001
EIN – Complex atypical	0.17	−0.86 to 1.20	0.74
EIN – Carcinoma	−2.46	−5.02 to 0.10	0.18

Notes. Overall ANOVA: $F(4,310)=61.33$, $p<0.0001$, $\eta^2=0.44$. Groups: Simple; Complex (no atypia); Complex atypical; EIN; Carcinoma.

Table S4. Spearman correlations between individual clinical factors and ordered histopathological severity

Predictor	Spearman ϱ	p-value
Obesity	0.184	0.0010
Hypertension	0.154	0.0061
Diabetes	0.125	0.0268
History of non-atypical hyperplasia	−0.139	0.0137
Oral contraceptives (long-term)	−0.126	0.0255
Infertility	0.034	0.56
Fibroids/Polyps	−0.061	0.29
Fibrocystic breast disease	−0.022	0.70
PCOS	−0.019	0.75
Estrogen-producing ovarian tumor	−0.028	0.64
Estrogen replacement therapy	0.031	0.60

Notes. Positive ϱ indicates higher severity with presence of the factor; negative ϱ indicates inverse association. Two-sided tests.

Table S5. Correlations for composite indices (e.g., metabolic index) and sensitivity analyses

Metric	Coding/definition	Spearman ϱ	p-value
Endometrial thickness (mm)	Continuous (TVUS)	0.634	≈7.6×10^{-37}
Metabolic index	Sum of obesity + hypertension + diabetes (0–3)	0.253	5.6×10^{-6}
Overall risk-burden count	Sum of all recorded risk factors (0–11)	0.064	0.254
Age group	0 = 45–50; 1 = 51–55	−0.009	0.92

Notes. Severity coded ordinally: 0 = Simple; 1 = Complex (no atypia); 2 = Complex atypical; 3 = EIN; 4 = Carcinoma.
